# Unveiling the Power of Gut Microbiome in Predicting Neoadjuvant Immunochemotherapy Responses in Esophageal Squamous Cell Carcinoma

**DOI:** 10.34133/research.0529

**Published:** 2024-11-14

**Authors:** Le Liu, Liping Liang, YingJie Luo, Jimin Han, Di Lu, RuiJun Cai, Gautam Sethi, Shijie Mai

**Affiliations:** ^1^ Integrated Clinical Microecology Center, Shenzhen Hospital, Southern Medical University, Shenzhen, China.; ^2^ Department of Gastroenterology, Zhujiang Hospital, Southern Medical University, Guangzhou, China.; ^3^ Department of Gastroenterology and Hepatology, Guangzhou Key Laboratory of Digestive Diseases, Guangzhou Digestive Disease Center, Guangzhou First People’s Hospital, School of Medicine, South China University of Technology, Guangzhou, China.; ^4^ Department of Thoracic Surgery, Nanfang Hospital, Southern Medical University, Guangzhou, China.; ^5^ School of Life Sciences, Tsinghua University, Beijing, China.; ^6^ Department of Pharmacology, Yong Loo Lin School of Medicine, National University of Singapore, Singapore, Singapore.

## Abstract

The role of the gut microbiome in enhancing the efficacy of anticancer treatments like chemotherapy and radiotherapy is well acknowledged. However, there is limited empirical evidence on its predictive capabilities for neoadjuvant immunochemotherapy (NICT) responses in esophageal squamous cell carcinoma (ESCC). Our study fills this gap by comprehensively analyzing the gut microbiome’s influence on NICT outcomes. We analyzed 16*S* rRNA gene sequences from 136 fecal samples from 68 ESCC patients before and after NICT, along with 19 samples from healthy controls. After NICT, marked microbiome composition changes were noted, including a decrease in ESCC-associated pathogens and an increase in beneficial microbes such as *Limosilactobacillus*, *Lacticaseibacillus*, and *Staphylococcus.* Baseline microbiota profiles effectively differentiated responders from nonresponders, with responders showing higher levels of short-chain fatty acid (SCFA)-producing bacteria such as *Faecalibacterium*, *Eubacterium_eligens_group*, *Anaerostipes*, and *Odoribacter*, and nonresponders showing increases in *Veillonella*, *Campylobacter*, *Atopobium*, and *Trichococcus.* We then divided our patient cohort into training and test sets at a 4:1 ratio and utilized the XGBoost-RFE algorithm to identify 7 key microbial biomarkers—*Faecalibacterium*, *Subdoligranulum*, *Veillonella*, *Hungatella*, *Odoribacter*, *Butyricicoccus*, and *HT002.* A predictive model was developed using LightGBM, which achieved an area under the receiver operating characteristic curve (AUC) of 86.8% [95% confidence interval (CI), 73.8% to 99.4%] in the training set, 76.8% (95% CI, 41.2% to 99.7%) in the validation set, and 76.5% (95% CI, 50.4% to 100%) in the testing set. Our findings underscore the gut microbiome as a novel source of biomarkers for predicting NICT responses in ESCC, highlighting its potential to enhance personalized treatment strategies and advance the integration of microbiome profiling into clinical practice for modulating cancer treatment responses.

## Introduction

Esophageal cancer ranks as the sixth most prevalent malignancy in China, and it stands as the fourth leading cause of cancer-related mortality nationally. Notably, esophageal squamous cell carcinoma (ESCC) accounts for over 90% of these cases [1,2]. The conventional therapeutic regimen for locally advanced ESCC typically comprises neoadjuvant chemoradiotherapy followed by minimally invasive esophagectomy [3], with neoadjuvant chemotherapy (NCT) particularly favored in regions such as China and Japan [4,5]. Although these treatments are associated with enhanced long-term survival, the persistence of high recurrence rates and only modest improvements in overall survival underscore the need for innovative therapeutic strategies. Emerging evidence from the CheckMate577 trial highlights the significant extension of median disease-free survival provided by postoperative adjuvant immunotherapy following neoadjuvant chemoradiotherapy and esophagectomy, up to 11.0 months [6]. Similarly, in the randomized, open-label, phase 3 ATTRACTION-3 study, nivolumab enhanced survival compared to chemotherapy in patients with advanced, previously treated ESCC [7]. Furthermore, integrating immunotherapy with NCT has demonstrated potential in locally advanced ESCC, achieving favorable pathological complete response (pCR) rates and manageable toxicity [8,9]. This integration suggests that neoadjuvant immunochemotherapy (NICT) could represent a promising advancement in the treatment landscape of ESCC, offering potential improvements in patient outcomes. pCR following neoadjuvant therapy is increasingly recognized as a crucial predictor of long-term survival across various cancer types [10,11]. Successfully achieving pCR after neoadjuvant therapy significantly enhances patient outcomes. In contrast, the prognosis for patients who do not achieve pCR may be less favorable than for those undergoing surgery alone, primarily due to the complications and toxicity associated with neoadjuvant therapy [12]. It is noteworthy that not all patients with ESCC respond to NICT. This observation prompts a crucial inquiry into the predictive markers and underlying mechanisms that could determine the responsiveness of ESCC patients to NICT.

There is growing evidence that polymorphic variability in individual microbiomes significantly influences cancer phenotypes and therapy responses. In addition to their role in tumor development, microbiotas are increasingly recognized for their ability to modulate therapy efficacy and toxicity. This modulation occurs through various mechanisms, including influencing drug pharmacokinetics, altering the host’s metabolic environment, or changing the composition of the tumor milieu [13–15]. Interestingly, studies have shown that antibiotic treatment during anti-PD-1/PD-L1 therapy or combined anti-CTLA-4 and PD-1/PD-L1 therapy in advanced esophagogastric cancer did not result in the adverse effects commonly seen in cases of lung cancer, renal cancer, or melanoma [16,17]. Thus, the impact of the gut microbiome on NICT in ESCC appears to be distinct from other tumor types and warrants further investigation. Patients with ESCC often exhibit reduced gut microbiota diversity and richness, characterized by a decrease in *Firmicutes* and an increase in *Enterobacteriaceae* and *Bacteroides* [16,18,19]. However, investigations into the shifts in gut microbiome dynamics during NICT and their correlation with therapeutic effectiveness are still in their infancy. In this study, we systematically collected fecal samples from patients diagnosed with ESCC at 2 critical time points: before and after undergoing NICT. The primary objective was to assess the correlations between the longitudinal changes observed in the gut microbiota and the pathologic responses across the treatment duration. By analyzing these temporal microbiota shifts, we aim to uncover potential microbial markers that might predict therapeutic outcomes. Additionally, this study seeks to explore the mechanisms by which variations in the microbiome could influence the efficacy of NICT, potentially offering insights into personalized therapeutic strategies based on individual microbiota profiles.

## Results

### Distinct microbial patterns and functional pathways in ESCC patients compared to healthy individuals

**Fig. 1.**
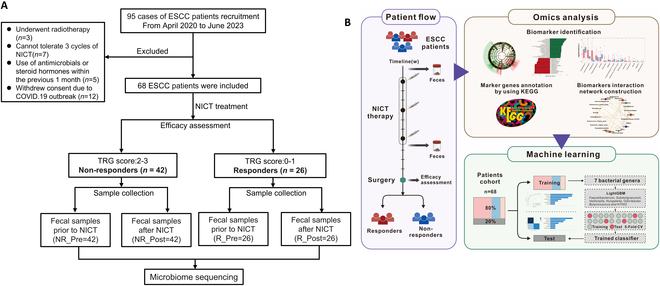
Flow chart. (A) Screening process and study design for the ESCC cohort. (B) Workflow overview for the microbiomic strategy implemented in this study.

**Fig. 2.**
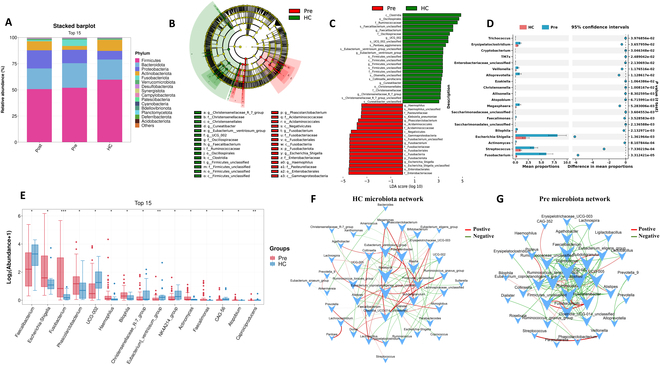
The gut microbiome in patients with ESCC significantly differed from that in healthy individuals. (A) Comparative stacked bar plot illustrating the phylum-level bacterial composition in fecal samples from ESCC patients before NICT (*n* = 68), after NICT (*n* = 68), and from HC (*n* = 19). (B) The taxonomic cladogram was generated based on the LEfSe and LDA scores. Bacteria taxa enriched in ESCC patient group (red) and control group (green). (C) Histogram showing the differentially abundant taxa between ESCC patients (red) and controls (green), analyzed by LEfSe. All listed taxa were significantly (Kruskal–Wallis test, *P* < 0.05, LDA score > 3) enriched for their respective groups. p, phylum; c, class; o, order; f, family; g, genus; s, species. (D) Differential relative abundances of top 20 genera by the STAMP analysis. (E) Box plot showing the relative abundances of the top 15 differentially abundant taxa in ESCC (red) and HC (blue) groups, identified using Mann–Whitney *U* test and adjusted by FDR correction. *P < 0.05, **P < 0.01, ***P < 0.001. (F) Microbial cooccurrence network deduced using Spearman rank correlations, based on samples from HC. Only the top 50 bacterial taxa by abundance and statistically significant connections (*P* < 0.05) with a magnitude greater than 0.4 (positive correlation, red edges) or less than −0.4 (negative correlation, green edges) are displayed. Each node is labeled at the genus level, and the size of node is proportional to corresponding abundances. (G) Microbial cooccurrence network based on samples from patients with ESCC. HC, healthy controls; Pre, ESCC patients before NICT therapy; Post, ESCC patients after NICT therapy.

The inclusion and exclusion criteria for patient selection, as well as the methodology of the study, are illustrated in the flowchart provided in Fig. 1A and B. To explore the microbiome dynamics in ESCC, we first compared the microbiome profiles between ESCC patients and healthy controls (HCs). Our analysis showed that *Firmicutes*, *Bacteroidetes*, *Proteobacteria*, and *Actinobacteria* were dominant across all samples, identifying these phyla as the microbial backbone in both groups (Fig. 2A). Through linear discriminant analysis effect size (LEfSe) analysis, we identified significant disparities in microbial populations, observing higher occurrences of *Haemophilus*, *Phascolarctobacterium*, *Fusobacterium*, and *Escherichia_Shigella* in ESCC patients, and a predominance of *Faecalibacterium*, *UCG_002*, *Eubacterium ventriosum_group*, *Firmicutes_unclassified*, *Cuneatibacter*, and *Christensenellaceae_R_7_group* in healthy individuals (Fig. 2B and C). Subsequent detailed comparisons using STAMP analysis revealed also significant differences; specifically, 22 genera including *Fusobacterium*, *Escherichia-Shigella*, *Bilophila*, *Erysipelatoclostridium*, *Veillonella*, *Alloprevotella*, *Megasphaera*, *Streptococcus*, *Faecalimonas*, *Actinomyces*, and others were significantly elevated in the ESCC cohort compared to the HC group (Fig. 2D). These results align with those obtained from differential analysis based on the Mann–Whitney *U* test (Fig. 2E). In the study of microbiota co-occurrence networks, unlike the homogeneous interactions within the microbiota of HC, the gut microbiota of ESCC patients exhibited more heterogeneity and negative regulatory relationships. Notably, in ESCC, the central bacterium *Coprococcus* was surrounded by short-chain fatty acid (SCFA)-producing bacteria such as *Faecalibacterium*, *Eubacterium_coprostanoligenes_group_unclassified*, and *Ruminococcaceae_unclassified*. This arrangement highlights the destructive role of *Coprococcus* in colonic health and its potential pro-tumorigenic effects. Conversely, in HC, the central bacterium *Roseburia* was observed, which is associated with cancer-suppressive activities (Fig. 2F and G).

**Fig. 3.**
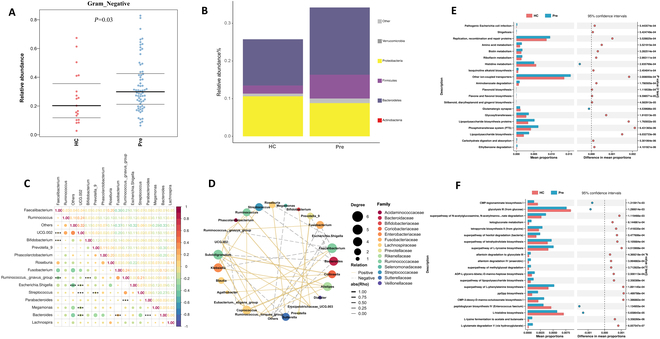
Functional analysis of the gut microbiome in ESCC patients. (A) BugBase is used to analyze the gut bacterial phenotypes. Scatterplots showed the phenotype as gram-negative bacteria from each group. The relative abundances of the gram-negative bacteria were higher in ESCC patients (*P* = 0.03). (B) Stacked bar plot of the gram-negative bacteria at the phylum level in fecal samples from ESCC patients and controls. (C) Correlation heatmap of the top 15 abundant bacterial genera in HC and ESCC patients. The size of the circle within each cell indicates the magnitude of correlation between 2 genera, with the color scale ranging from +1 (strong positive correlation) to −1 (strong negative correlation). The numbers displayed within the cells provide the exact values of the correlation coefficients. (D) Correlation network among the top 30 most abundant bacterial genera in HC and ESCC patients. Colors represent the family to which each genus belongs. Solid lines denote positive correlations, while dashed lines indicate negative correlations between genera. The thickness of each line correlates with the strength of the Spearman’s correlation coefficient (Rho), and only statistically significant correlations (*P* < 0.05) are displayed. (E) Comparative analysis of predicted KEGG level 3 functional categories between HC and ESCC patients using PICRUSt. (F) Comparative analysis of predicted MetaCyc pathways of the gut microbiome between HC and ESCC patients using PICRUSt.

In terms of bacterial phenotype predictions, our study noted an increase in gram-negative bacteria in ESCC patients compared to healthy individuals (Fig. 3A and B). This observation is significant given the pathogenic propensity of these bacteria. The correlation heatmap and association network (Fig. 3C and D) illustrate that beneficial bacteria such as *Faecalibacterium*, *Bifidobacterium*, and *Eubacterium_eligens_group* demonstrate strong positive interactions, hinting at cooperative benefits to the host’s well-being. However, significant negative correlations exist between *Faecalibacterium* and pathogens like *Fusobacterium* and *Klebsiella.* Utilizing the PICRUSt computational tool to predict functional profiles, we identified significant differences in the Kyoto Encyclopedia of Genes and Genomes (KEGG) signaling pathways at level 3 between the groups. Specifically, the ESCC group demonstrated a notable increase in the functional genes of the gut microbiota involved in “lipopolysaccharide biosynthesis” and “pathogenic *Escherichia coli* infection” (Fig. 3E). Additionally, when comparing healthy individuals to ESCC patients prior to NICT, we observed substantial variations in the MetaCyc metabolic pathways. These included pathways potentially influential in cancer pathology, such as the “superpathway of tetrahydrofolate biosynthesis” and “CMP-3-deoxy-d-manno-octulosonate biosynthesis I” (Fig. 3F).

### Impact of NICT therapy on gut microbiome dynamics and therapeutic response

**Fig. 4.**
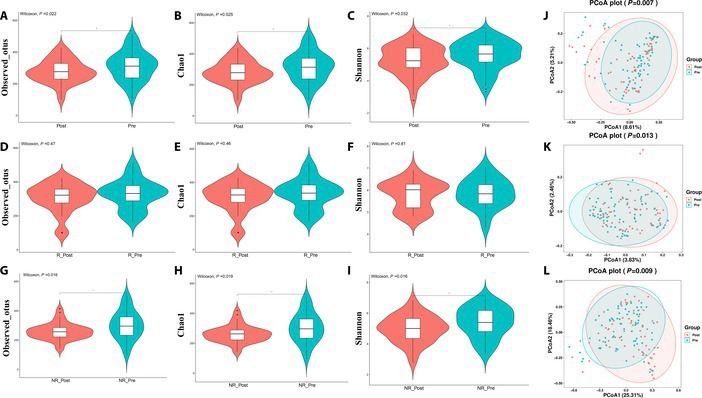
Alterations in gut microbiome diversity during NICT therapy. Alpha diversity was estimated using the observed operational taxonomic units (OTUs) (A, D, and G), Chao1 (B, E, and H), and Shannon diversity index (C, F, and I) and analyzed by Wilcoxon rank sum test and represents *P* < 0.05. Beta diversity analysis was performed by using the Bray–Curtis (J)-, Jaccard (K)-, and weighted UniFrac (L)-based PCoA.

After establishing baseline gut microbiome profiles in ESCC patients, we analyzed fecal samples before and after NICT therapy to explore microbiome dynamics in response to treatment. Initial findings showed that microbial diversity was significantly higher in samples collected before NICT therapy compared to after therapy (Fig. 4A to C), a pattern that was almost consistent across both responder and nonresponder subgroups. Notably, nonresponders exhibited a more pronounced reduction in microbial diversity, as evidenced by significant drops in observed species and the Chao1 index, a contrast not as evident among responders (Fig. 4D to I). Principal coordinate analysis (PCoA) using Bray–Curtis, Jaccard, and weighted UniFrac metrics further demonstrated distinct clustering by sampling time points, highlighting the temporal impact of NICT therapy on the gut microbiome (*P* = 0.007, *P* = 0.013, and *P* = 0.009, respectively) (Fig. 4J to L).

**Fig. 5.**
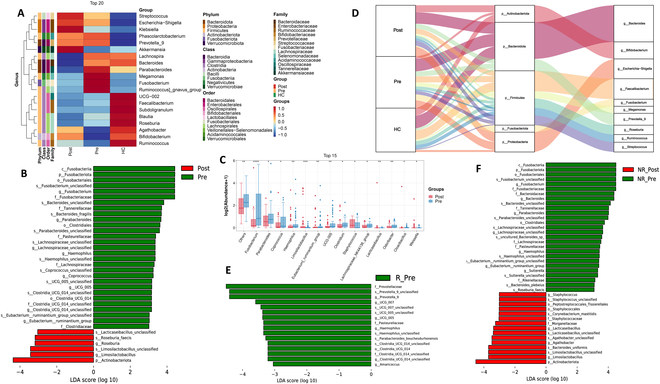
Gut microbiome variations over the course of NICT therapy associated with therapeutic response. (A) Heatmap displaying the top 20 bacterial genera abundance at the genus level across different groups. (B) Histogram showing the differentially abundant taxa between Post group (red) and Pre group (green), analyzed by LEfSe. All listed taxa were significantly (Kruskal–Wallis test, *P* < 0.05, LDA score > 3) enriched for their respective groups. (C) Boxplot displays the top 15 differential abundances in ESCC patients before and after NICT treatment, determined through Mann–Whitney *U* test and adjusted by FDR correction. Significance is denoted as *P < 0.05, **P < 0.01, ***P < 0.001. (D) Sankey diagram of the top 10 bacterial genera abundance in HC and ESCC patients before and after NICT treatment. (E) Histogram showing the differentially abundant taxa in responder ESCC patients before and after NICT treatment. (F) Histogram showing the differentially abundant taxa in nonresponder ESCC patients before and after NICT treatment, analyzed by LEfSe. All listed taxa were significantly (Kruskal–Wallis test, *P* < 0.05, LDA score > 3) enriched for their respective groups.

A heatmap of the top 20 bacterial genera revealed distinct clustering between groups. Genera like *Faecalibacterium*, *Blautia*, and *Bifidobacterium* dominated in the HC cohort, suggesting a stable, health-associated microbial environment. Conversely, the Pre group exhibited higher levels of potentially dysbiotic genera such as *Fusobacterium*, *Megamonas*, and *Escherichia-Shigella.* The Sankey diagram showed microbial composition shifts after treatment, with genera like *Bifidobacterium* and *Ruminococcus* partially reverting to patterns similar to those of HCs (Fig. 5A and D). Differential analysis using LEfSe and Mann–Whitney *U* test indicated significant microbial changes after NICT therapy. There was a decrease in genera such as *Fusobacterium* and *Haemophilus*, while *Limosilactobacillus*, *Lacticaseibacillus*, and *Staphylococcus* increased in the posttreatment group (Fig. 5B and C). Notably, increases in *Limosilactobacillus* and *Lacticaseibacillus* were pronounced among nonresponders, suggesting a paradoxical rise in these beneficial bacteria in patients unresponsive to NICT (Fig. 5E and F).

**Fig. 6.**
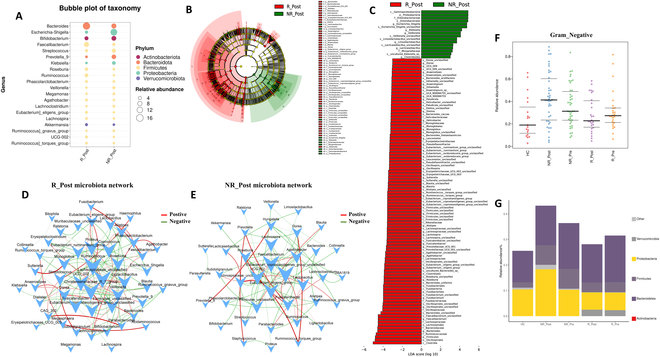
Decoding the gut microbiome’s response to NICT therapy in ESCC through microbial abundance, network interactions, and phenotypes. (A) Bubble plot visualizing the top 20 bacterial genera abundance in R_Post group and NR_Post group. (B) The taxonomic cladogram was generated based on the LEfSe and LDA scores. Bacteria taxa enriched in R_Post group (red) and NR_Post group (green). (C) Histogram showing the differentially abundant taxa between R_Post group (red) and NR_Post group (green), analyzed by LEfSe. All listed taxa were significantly (Kruskal–Wallis test, *P* < 0.05, LDA score > 3) enriched for their respective groups. (D) Microbial cooccurrence network deduced using Spearman rank correlations, based on samples from R_Post group. Only the top 50 bacterial taxa by abundance and statistically significant connections (*P* < 0.05) with a magnitude greater than 0.4 (positive correlation, red edges) or less than −0.4 (negative correlation, green edges) are displayed. Each node is labeled at the genus level, and the size of node is proportional to corresponding abundances. (E) Microbial cooccurrence network based on samples from NR_Post group. (F) BugBase is used to analysis the gut bacterial phenotypes. Scatterplots showed the phenotype as gram-negative bacteria from each group. (G) Stacked bar plot of the gram-negative bacteria at the phylum level in fecal samples from each group. R_Post, responders after NICT therapy; NR_Post, nonresponders after NICT therapy; R_Pre, responders before NICT therapy; NR_Pre, nonresponders before NICT therapy.

Significant differences in alpha diversity were observed between the responder and nonresponder groups after therapy (Fig. S1A to C). PCoA using Bray–Curtis, Jaccard, and weighted UniFrac distances successfully distinguished the groups after therapy (Fig. S1G to I). The bubble plot showed enrichment of beneficial genera like *Bifidobacterium* and *Faecalibacterium* in responders after therapy, linking these bacteria to positive therapeutic outcomes. In contrast, nonresponders after therapy exhibited significant enrichment of potentially dysbiotic genera such as *Escherichia-Shigella*, *Veillonella*, and *Klebsiella* (Fig. 6A to C). The co-occurrence network for responders illustrated a tightly knit community of beneficial bacteria, primarily involved in synthesizing SCFAs, with genera such as *Streptococcus*, *Dorea*, and *Roseburia* playing central roles. In contrast, the network for nonresponders featured a notable presence of genera associated with dysbiosis and adverse health outcomes, potentially involved in indole synthesis, such as *Alistipes* and *Agathobacter* (Fig. 6D and E). Additionally, phenotypic analysis using BugBase indicated a trend where NICT reduced the prevalence of gram-negative bacteria in responders, while an increase was observed in nonresponders (Fig. 6F and G).

### Predictive potential of the gut microbiome in NICT outcomes for ESCC

**Fig. 7.**
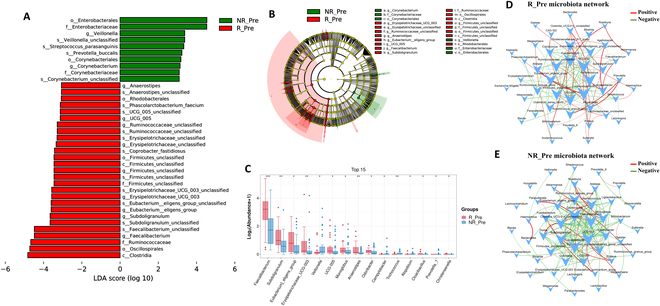
Baseline gut microbiome correlated with therapeutic response to NICT therapy in ESCC patients. (A) Histogram showing the differentially abundant taxa between R_Pre group (red) and NR_Pre group (green), analyzed by LEfSe. All listed taxa were significantly (Kruskal–Wallis test, *P* < 0.05, LDA score > 3) enriched for their respective groups. (B) Cladogram showing the differentially abundant taxa between R_Pre group (red) and NR_Pre group (green), analyzed by LEfSe. (C) Boxplot illustrates the top 15 differential abundances between the R_Pre group and the NR_Pre group, analyzed using the Mann–Whitney *U* test with subsequent FDR correction. Significance levels are indicated as *P < 0.05, **P < 0.01, and ***P < 0.001. (D) Microbial cooccurrence network deduced using Spearman rank correlations, based on samples from R_Pre group. Only the top 50 bacterial taxa by abundance and statistically significant connections (*P* < 0.05) with a magnitude greater than 0.4 (positive correlation, red edges) or less than −0.4 (negative correlation, green edges) are displayed. Each node is labeled at the genus level, and the size of node is proportional to corresponding abundances. (E) Microbial cooccurrence network based on samples from NR_Pre group.

Building on our insights, we hypothesized that the gut microbiome could serve as a predictive biomarker for responses to NICT in ESCC patients. We conducted a comparative microbial analysis between responder and nonresponder cohorts before NICT therapy, summarizing their baseline demographics and clinical features in Table 1. Our findings revealed no significant differences in alpha diversity between the responder and nonresponder groups (Fig. S1D to F). Additionally, PCoA using Bray–Curtis, Jaccard, and weighted UniFrac distances failed to distinguish the groups at baseline (Fig. S1J to L). Employing LEfSe analysis, we identified distinct microbial signatures: Responders were dominated by beneficial microbes such as *Anaerostipes*, *Erysipelotrichaceae_UCG_003*, *Subdoligranulum*, *Faecalibacterium*, *Eubacterium_eligens_group*, and *Phascolarctobacterium_faecium.* In contrast, nonresponders showed significant enrichment of potentially pathogenic bacteria like *Enterobacteriaceae*, *Corynebacteriaceae*, *Veillonella*, *Corynebacterium*, *Streptococcus_parasanguinis*, and *Prevotella_buccalis* (Fig. 7A and B). Confirmatory Mann–Whitney *U* tests with false discovery rate (FDR) correction demonstrated a higher relative abundance of beneficial bacteria in responders (Fig. 7C).

**Table 1.** Baseline characteristics of the patients with ESCC enrolled in this study. Clinical staging before NICT therapy was performed according to the AJCC (eighth edition). *P* values < 0.05 are in bold.

Variable	Items	Overall (*n* = 68)	Group NR (*n* = 42)	Group R (*n* = 26)	*P*
Gender, *n* (%)	Female	15 (22.059)	12 (28.571)	3 (11.538)	0.100
	Male	53 (77.941)	30 (71.429)	23 (88.462)	
Smoking, *n* (%)	Frequently	30 (44.118)	19 (45.238)	11 (42.308)	0.807
	Never	25 (36.765)	16 (38.095)	9 (34.615)	
	Occasionally	13 (19.118)	7 (16.667)	6 (23.077)	
Drinking, *n* (%)	Frequently	29 (42.647)	19 (45.238)	10 (38.462)	0.650
	Never	32 (47.059)	18 (42.857)	14 (53.846)	
	Occasionally	7 (10.294)	5 (11.905)	2 (7.692)	
Tumor location, *n* (%)	Low	20 (29.412)	13 (30.952)	7 (26.923)	0.723
	Middle-high	48 (70.588)	29 (69.048)	19 (73.077)	
Surgical method, *n* (%)	Mckeown	51 (75.000)	30 (71.429)	21 (80.769)	0.687
	Sweet	10 (14.706)	7 (16.667)	3 (11.538)	
	lvor Lewis	7 (10.294)	5 (11.905)	2 (7.692)	
Clinical T stage, *n* (%)	T2	1 (1.471)	0 (0.000)	1 (3.846)	0.228
	T3	52 (76.471)	31 (73.810)	21 (80.769)	
	T4	15 (22.059)	11 (26.190)	4 (15.385)	
Clinical N stage, *n* (%)	N0	20 (29.412)	12 (28.571)	8 (30.769)	0.653
	N1	16 (23.529)	12 (28.571)	4 (15.385)	
	N2	25 (36.765)	14 (33.333)	11 (42.308)	
	N3	7 (10.294)	4 (9.524)	3 (11.538)	
Clinical staging before NICT therapy, *n* (%)	II	16 (23.529)	9 (21.429)	7 (26.923)	0.651
	III	32 (47.059)	19 (45.238)	13 (50.000)	
	IVA	20 (29.412)	14 (33.333)	6 (23.077)	
Age, year, median [IQR]	nan	63.000 [54.000, 67.000]	61.000 [54.000, 67.000]	63.000 [55.000, 67.000]	0.714
BMI, kg/m^2^, mean (±SD)	nan	21.997 ± 3.382	21.467 ± 3.346	22.854 ± 3.263	0.103
TRG score, median [IQR]	nan	2.000 [1.000, 3.000]	3.000 [2.000, 3.000]	0.000 [0.000, 1.000]	**<0.001**

NR, nonresponders; R, responders

**Fig. 8.**
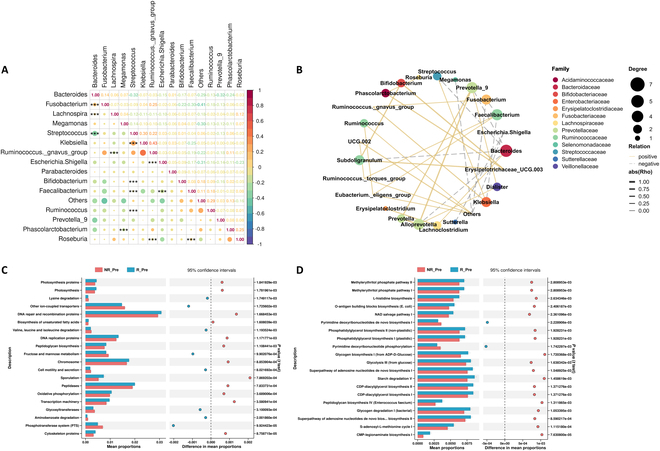
Microbial network and pathway signatures predicting NICT response in ESCC. (A) Correlation heatmap of the top 15 abundant bacterial genera in R_Pre and NR_Pre groups. (B) Correlation network among the top 30 most abundant bacterial genera in R_Pre and NR_Pre patients. Colors represent the family to which each genus belongs. Solid lines denote positive correlations, while dashed lines indicate negative correlations between genera. The thickness of each line correlates with the strength of the Spearman’s correlation coefficient (Rho), and only statistically significant correlations (*P* < 0.05) are displayed. (C) Comparative analysis of predicted KEGG level 3 pathways between R_Pre and NR_Pre in ESCC patients. (D) Predicted MetaCyc pathway abundance comparison between R_Pre and NR_Pre groups in ESCC Patients.

In the R_Pre co-occurrence network, beneficial SCFA-producing bacteria formed a cohesive cluster, exhibiting positive regulatory relationships. Conversely, the NR_Pre network showed a higher prevalence of taxa associated with negative health outcomes, such as *Coprococcus*, *Collinsella*, and *Bilophila*, suggesting a dysbiotic state that could negatively impact NICT therapy response (Fig. 7D and E). The heatmap and network diagrams (Fig. 8A and B) highlighted strong positive correlations between *Faecalibacterium* and beneficial genera like *Ruminococcus torques* group, alongside negative correlations with pathogens such as *Fusobacterium* and *Klebsiella*, indicating potential competitive interactions or ecological segregation. Microbial community function analysis revealed that pathways critical for cell repair and growth, such as DNA repair and recombination proteins, DNA replication proteins, oxidative phosphorylation, and biosynthesis of unsaturated fatty acids, were more prevalent in responders. In contrast, nonresponders exhibited enrichment in pathways like lysine degradation and phosphotransferase system, which may negatively influence immune responses (Fig. 8C). Additionally, responders showed enrichment in metabolic pathways beneficial to commensal bacteria, such as l-histidine biosynthesis and the methylerythritol phosphate pathway (Fig. 8D). These findings illuminate distinct functional profiles of the pretreatment microbiome, underlining the importance of microbiome composition in influencing therapy outcomes.

### Optimizing NICT outcomes in ESCC using microbiome-derived predictive models and machine learning

**Fig. 9.**
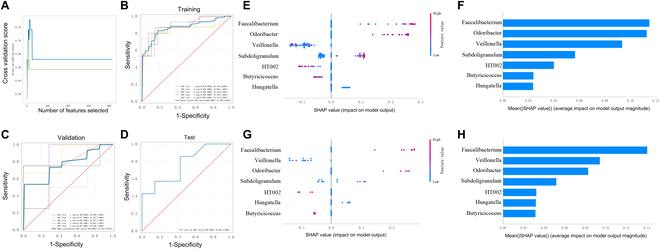
Gut microbiome-based classifier for predicting NICT therapy response in ESCC. (A) Characteristic variable screening based on XGBoost-RFE. (B) ROC curve for NICT response prediction model in training set. (C) Validation ROC curve for NICT response prediction model. (D) Testing ROC curve for predictive model validation on independent dataset. (E) SHAP value plots for NICT therapy prediction model in training set. (F) Ranking of bacterial features by mean absolute SHAP values in the training set. (G) SHAP value plots for NICT therapy prediction model in testing set. (H) Ranking of bacterial features by mean absolute SHAP values in the testing set.

Our study seeks to harness the potential of the gut microbiome as predictive biomarkers for NICT response in ESCC through advanced machine learning techniques. We divided the study cohort into training and test sets in a 4:1 ratio. Using the extreme gradient boosting (XGBoost)–recursive feature elimination (RFE) algorithm on the training set, we identified 7 bacterial taxa—*Faecalibacterium*, *Subdoligranulum*, *Veillonella*, *Hungatella*, *Odoribacter*, *Butyricicoccus*, and *HT002*—that showed promise as predictors of NICT response (Fig. 9A). To refine our predictive capabilities, we employed the LightGBM algorithm on the training set, enhanced by rigorous 5-fold cross-validation. This method successfully distilled these markers into an optimal classifier set, which was then evaluated to yield receiver operating characteristic (ROC) curves for both training and validation phases. The training phase demonstrated a compelling average area under the curve (AUC) of 86.8% [95% confidence interval (CI), 73.8% to 99.4%], with the validation phase maintaining model reliability at an AUC of 76.8% (95% CI, 41.2% to 99.7%) (Fig. 9B and C). When tested against an independent set, the model upheld its discriminative accuracy with a test AUC of 76.5% (95% CI, 50.4% to 100%) (Fig. 9D).

Based on the data in Table 2, applying an optimal decision threshold of 0.445 determined through ROC curve analysis, our predictive model achieved high specificity and positive predictive value (PPV) in both the training and validation cohorts. In the training cohort, the model demonstrated an AUC of 0.868, accuracy of 83.3%, sensitivity of 73.7%, specificity of 88.6%, PPV of 79.8%, and negative predictive value (NPV) of 86.2%. The validation cohort showed similar results, with an AUC of 0.768, accuracy of 72.5%, sensitivity of 58.3%, specificity of 80.0%, and PPV of 68.6%. These metrics underscore the model’s robustness in effectively predicting the NICT therapy response. In the testing cohort, using a cutoff of 0.420, the model maintained good predictive ability with an AUC of 0.765, accuracy of 64.3%, sensitivity of 57.1%, specificity of 71.4%, and PPV of 66.7%. Although the performance metrics are slightly lower in the testing cohort, the model still effectively distinguished between responders and nonresponders. Overall, our predictive model performed well across all cohorts, accurately distinguishing between responders and nonresponders with high specificity and PPV. These results validate the model’s effectiveness and highlight its potential clinical utility in screening and identifying suitable candidates for NICT therapy. Furthermore, shapley additive explanation (SHAP) value analysis revealed that bacterial genera such as *Faecalibacterium*, *Odoribacter*, and *Veillonella* were key to the accuracy of the LightGBM predictive model for NICT therapy in ESCC, showcasing their significant contribution to model performance (Fig. 9E to H). Even with varied impacts in the test set, the consistent significance of *Odoribacter*, *Veillonella*, and *Subdoligranulum* highlights nuanced roles these microbes play across different dataset subsets, suggesting their integral involvement in therapy response dynamics. These findings affirm the value of integrating machine learning with microbial profiling to advance personalized treatment approaches in oncology, showcasing the microbiome as both a predictor and a modifiable target in personalized cancer therapy.

**Table 2.** Performance of the microbial prediction classifiers and clinical testing for patients

	Value in classifier (95% CI)		
	Training cohort	Validation cohort	Testing cohort
AUC	0.868 (0.738–0.994)	0.768 (0.412–0.997)	0.765 (0.504–1.000)
Cutoff	0.445 (0.332–0.559)	0.445 (0.332–0.559)	0.420
ACC	0.833 (0.800–0.866)	0.725 (0.578–0.872)	0.643
SE	0.737 (0.695–0.779)	0.583 (0.397–0.770)	0.571
SP	0.886 (0.818–0.954)	0.800 (0.590–1.000)	0.714
PPV	0.798 (0.685–0.911)	0.686 (0.433–0.938)	0.667
NPV	0.862 (0.849–0.874)	0.776 (0.693–0.859)	0.625
F1 score	0.759 (0.728–0.790)	0.607 (0.416–0.798)	0.615

ACC, accuracy; SE, sensitivity; SP, specificity; PPV, positive predictive value; NPV, negative predictive value

## Discussion

NICT is fast becoming a pivotal strategy in oncology, particularly designed to reduce tumor load and size, thereby enhancing the prospects of complete surgical resection. Existing studies have validated NICT’s effectiveness in significantly enhancing pCR rates, increasing surgical resection rates, and substantially reducing toxicity associated with esophageal cancer treatments [20,21]. Despite these advances, predicting responses to NICT in ESCC remains a formidable challenge due to the lack of widely accepted, sensitive, and reliable biomarkers. Our study advances this field by introducing a gut microbiome-based classifier using noninvasive fecal sampling. This classifier, developed through the LightGBM algorithm, is distinguished by its precision and efficiency, offering a practical tool for clinical settings. Demonstrating high specificity and predictive value, it identifies potential responders to NICT, significantly enhancing personalized treatment strategies in ESCC.

ESCC is the most common histological type of esophageal cancer, accounting for over 90% of cases. Compared to healthy individuals and patients with esophageal adenocarcinoma, those with ESCC exhibit a distinct gut microbiome community. In the study, we first provide substantial evidence of specific pathogenic bacteria like *Fusobacterium*, *Escherichia-Shigella*, *Faecalimonas*, *Bilophila*, *Actinomyces*, and *Bacteroides fragilis* to better understand the gut microbiome’s complex role in ESCC development. These organisms are more common in patients than HCs and contribute to cancer progression in numerous ways. Previous research has established a connection between *Fusobacterium* and the progression and prognosis of ESCC. *Fusobacterium nucleatum* has been detected in both colorectal cancer tissues and fecal samples, influencing treatment responses by targeting Toll-like receptor 4 (TLR4) and MyD88 immune pathways and specific microRNAs to activate autophagy, thus serving as a biomarker for colorectal cancer [22]. Although primarily found in the oral cavity, *F. nucleatum* has been confirmed in the gut and linked to colorectal cancer by studies at the BC Cancer Research Centre and the Broad Institute [23]. Furthermore, Yamamura et al. [24] showed that *F. nucleatum* could facilitate tumor invasion by activating CCL20, impacting esophageal cancer. However, most studies focus on tumor biopsies rather than fecal samples, raising questions about the comparability of findings from different sample types, and underscoring the need for more extensive validation. *Escherichia-Shigella*, a group of gut microbiota bacteria, has been associated with the progression of ESCC. These bacteria can promote inflammation and immune evasion, disrupting normal cellular functions and altering the local immune environment crucial for tumor development. Additionally, an increase in *Escherichia-Shigella* may cause dysbiosis, a microbial imbalance linked to cancer progression [25]. *Bilophila*, a sulfite-reducing bacterium in the human gut, metabolizes sulfonates to produce hydrogen sulfide, associated with inflammatory conditions and colorectal cancer. By weakening the intestinal barrier and fostering inflammation, *Bilophila* may influence tumorigenesis, highlighting its significant role in microbiome-associated diseases [26]. Li et al. [27] also utilized the 16*S* ribosomal RNA (rRNA) method to identify disparities in the fecal microbiota of ESCC patients, notably observing a significant depletion of *Prevotella* compared to healthy individuals. Furthermore, Liu’s team conducted similar analyses and noted a potential link between *Prevotella* and lymph node metastasis in ESCC, proposing *Prevotella* as a potential independent prognostic marker [28]. They reported that *Prevotella* primarily contributes to the initiation of inflammation by prompting epithelial cells to release pro-inflammatory cytokines, thereby inducing immune pathological alterations that facilitate the onset and advancement of ESCC. In contrast, healthy people’s microbiomes contain beneficial microbes like *Faecalibacterium*, which produce SCFAs and reduce inflammation, *Eubacterium_ventriosum_group*, *Cuneatibacter*, and *Christensenellaceae_R_7_group*, which maintain gut barrier integrity and modulate immune responses. This sharp contrast shows that microbiome changes can be used to produce tailored cancer treatments and prevention. The correlation heatmap and association network among different bacterial genera reveal a dynamic ecosystem characterized by both supportive and competitive interactions. This vibrant and interdependent microbial network suggests a potential tug-of-war for dominance within the gut milieu. Beneficial bacteria, such as *Faecalibacterium*, *Bifidobacterium*, and *Eubacterium_eligens_group*, demonstrate strong positive interactions, suggesting cooperative benefits to the host’s well-being. However, significant negative correlations exist between *Faecalibacterium* and pathogens such as *Fusobacterium* and *Klebsiella.*

Advancements in surgical techniques, radiation targeting, chemotherapy regimens, and the inclusion of immunotherapy and targeted therapy have significantly improved survival and quality of life for ESCC patients. A multimodal treatment approach is now standard, enhancing outcomes. However, variability in response among similarly staged patients highlights the limitations of the Union for International Cancer Control/American Joint Committee on Cancer (UICC/AJCC) tumor-node-metastasis (TNM) staging system in predicting individual responses, emphasizing the need for personalized treatments. Biomarkers such as PD-L1 combined positive score, PD-L1 tumor proportion score, microsatellite instability, tumor mutation burden, and immune signatures are proposed to predict immunotherapy efficacy, but consensus is lacking, and their use in chemotherapy and radiotherapy is underexplored. Emerging evidence suggests the gut microbiome as a promising biomarker influencing responses to chemotherapy and immunotherapy. Research indicates that it modulates immune responses critical for immunotherapy, with distinct microbial profiles correlating with different treatment outcomes. Experiments involving fecal microbiota transplantation (FMT) in germ-free or antibiotic-treated mice have demonstrated that manipulating specific gut microbes can enhance the response to immunotherapy in these models [16,29]. Our analysis also revealed that NICT therapy reduced bacterial richness in ESCC patients, consistent with observations in exclusive immunotherapy studies. This reduction in microbial diversity can modulate host immune responses and potentially affect the success of cancer treatments. A diminished microbial diversity may impair the immune system’s ability to respond effectively to cancer cells, influencing therapeutic outcomes. After NICT therapy, we observed an increase in *Bifidobacterium*, a probiotic known for lactate production and pathogen suppression. The enrichment of *Bifidobacterium* suggests that the gut microbiota may adapt to engage the immune system against cancer cells, enhancing the therapeutic benefits of NICT therapy [19]. This indicates that certain beneficial bacteria can play a supportive role in modulating the immune response during cancer treatment. Our findings also showed a considerable reduction in microorganisms linked with poor ESCC outcomes after NICT therapy, bolstering the approach’s therapeutic efficacy. The decrease in harmful genera such as *Fusobacterium*, *Parabacteroides*, *Coprococcus*, and *Haemophilus* suggests that NICT therapy can alter the gut microbiome in a way that is conducive to better clinical responses. Interestingly, we found increases in *Lacticaseibacillus* and *Limosilactobacillus* in the nonresponder subgroup after therapy. This suggests complex interactions within the gut microbiome that may affect cancer treatment. The rise of these bacteria in nonresponders indicates that not all shifts in the microbiome are beneficial and that certain microbial changes may be associated with resistance to therapy. These observations underscore the intricate relationship between the gut microbiome and the host immune system during cancer treatment. Understanding these dynamics is crucial, as alterations in microbial diversity and composition can significantly impact therapeutic efficacy.

Significant research links *F. nucleatum* in ESCC tissues to poor prognosis. Shao et al. [30] reported distinct microbial profiles between ESCC tumors and adjacent tissues, with increased *Fusobacteria* in tumors and higher *Helicobacter* levels in nontumor tissues. *F. nucleatum*, associated with advanced tumor stages, emerges as a prognostic biomarker for ESCC. Yamamura et al. [24] detected *F. nucleatum* DNA in 23% of 325 samples, correlating its abundance with advanced tumor stages and reduced survival. Similarly, Li et al. [31] found elevated *F. nucleatum* levels in tumor tissues, linked to higher tumor and clinical stages, indicating its role in tumor progression. In colorectal and other cancers, *F. nucleatum* may reduce the efficacy of therapies like NICT and actively alter host responses, promoting cancer progression [32–34], underscoring the need for further study. *F. nucleatum*’s virulence factor, FadA adhesin, interacts with epithelial cell E-cadherin to activate the β-catenin pathway and enhance cellular proliferation, promoting tumor growth and metastasis [35]. It also circumvents the immune response by activating the T cell immunoglobulin and ITIM domain (TIGIT) checkpoint on immune cells with the Fap2 protein, reducing natural killer and tumor-infiltrating lymphocyte cytotoxicity [36]. It balances tumor cell apoptosis and autophagy via the TLR4/MyD88 signaling pathway, promoting chemoresistance to 5-fluorouracil and oxaliplatin [37]. In addition, the TLR4/nuclear factor κB (NF-κB) pathway activation by *F. nucleatum* increases BIRC3 expression, which inhibits apoptosis and improves cell survival during treatment stress [38]. *F. nucleatum* alters the tumor microenvironment by promoting immunosuppressive cells like myeloid-derived suppressor cells, tumor-associated neutrophils, and macrophages, leading to an inflammatory state that promotes tumor progression and immune evasion, characterized by elevated levels of pro-inflammatory cytokines like interleukin-1β (IL-1β), IL-6, and tumor necrosis factor-α (TNF-α). These complex relationships suggest that *F. nucleatum* may withstand NICT therapy and emphasize the need for specific therapies to inhibit these bacterial-mediated mechanisms.

Similarly, the role of SCFA-producing bacteria in modulating immune responses emphasizes the intricate balance between microbial metabolism and immune modulation, which is pivotal for enhancing the efficacy of immunotherapy. In the gut microbiome, SCFA-producing *Faecalibacterium*, *Butyricicoccus*, and *Odoribacter* promote cancer immunotherapy. Immunomodulatory SCFAs are produced by gut bacteria from dietary fibers, predominantly *Firmicutes.* It may alter immune checkpoint inhibitor (ICI) efficacy and side effects by increasing regulatory T cells (T_regs_) and reducing inflammation on myeloid cells and colonocytes via the GPR109a [39,40]. *Fecalibacterium* boosts T_regs_, IL-10, and IL-33, which maintain intestinal anti-inflammatory balance [41–43]. A lower initial CD4^+^ T_reg_ fraction increases anti-CTLA-4 treatment response. Gopalakrishnan et al. [44] discovered that *Faecalibacterium* boosted intratumoral, peripheral, and effector CD4^+^ T cells and cytokine levels, improving anti-PD-1 therapy. *Faecalibacterium* baseline increases can stabilize or aggravate stage III or IV cutaneous melanoma patients undergoing anti-PD-1 treatment [45]. Due to small sample sizes, diverse microbiota analysis methods, and different population demographics, immunotherapy response studies on significant microbial species may have variable results. Depending on SCFA content and host genetic background, SCFA-producing bacteria can be oncogenic or tumor suppressive, showing the gut microbiome’s complex impact on cancer and immunotherapy. Vancomycin, which lowers SCFA-producing bacteria, boosts immunotherapy but is balanced by butyrate’s influence on dendritic cell activities, highlighting the delicate balance between microbial metabolism and immunological modulation in cancer treatment [46]. ICI therapeutic efficacy and side effects can be improved by targeting the gut microbiota with SCFAs’ protection against ICI-induced colitis and other inflammatory disorders and developing techniques like FMT and prebiotic therapies. Dietary prebiotics that promote butyrate synthesis may minimize ICI-associated colitis, improve treatment outcomes, and increase ICI efficacy.

Due to the complexity and volume of microbiome data, advanced analytical methods like machine learning are essential [47,48]. We employed the LightGBM algorithm to identify therapeutically important microbial indicators within high-dimensional datasets. This approach not only demonstrates the algorithm’s capability to process complex data but also enhances our understanding of the microbiome’s role in cancer treatment. Our study reveals that gut microbiota compositions significantly influence NICT outcomes, emphasizing the microbiome’s critical role in treatment success. The classifier utilizing biomarkers such as *Faecalibacterium*, *Subdoligranulum*, *Veillonella*, *Hungatella*, *Odoribacter*, *Butyricicoccus*, and *HT002* achieved high predictive accuracy, with AUCs of 86.8% in the training cohort and 76.8% in the validation cohort, and maintained good performance in the testing cohort. These metrics underscore the model’s robustness and its potential clinical utility in effectively predicting NICT response. The application of machine learning in microbiome research adds significant value by enabling the identification of subtle patterns and interactions within complex microbial communities. Machine learning models can handle large, multidimensional datasets, uncovering predictive microbial markers that may not be apparent through traditional analysis methods. This capability is crucial for personalizing therapy strategies based on individual microbial profiles. Research consistently shows that the gut microbiota profoundly impacts cancer therapy responses, particularly in immunotherapies [16,18]. The interactions between the microbiota and the innate and adaptive immune systems modify immune cell activities and alter the tumor microenvironment’s immunological landscape, thereby affecting NICT efficacy [49]. Concurrently, studies on FMT demonstrate the microbiome’s potential to complement ICIs [50,51], illustrating its capacity to augment cancer treatment and support the integration of microbiome profiling in clinical practice.

Our study also underscores several limitations and indicates directions for future research. First, while we demonstrated a correlation between the microbiota and ESCC, we cannot determine causality from our observational data. The conclusions are also drawn from a specific patient population at our institution, potentially limiting their generalizability. To ensure broader applicability, further validation across diverse cohorts from various centers is required. Second, we did not analyze the impact of postoperative lifestyle factors such as smoking, drinking, and unhealthy dietary habits on tumor recurrence and the efficacy of NICT. Intervening in these risk factors can effectively reduce the risk of tumor recurrence, and their omission may affect the accuracy of our conclusions. Additionally, the variability in treatment outcomes may be influenced by different immunotherapy regimens, suggesting the necessity to explore the effects of various treatments more thoroughly. Furthermore, while our genus-level analysis offers a stable overview, it may miss finer interactions at the species level, such as the specific role and mechanisms of *F. nucleatum* in ESCC. Although we found that *Fusobacterium* is enriched in the gut of ESCC patients and is related to NICT efficacy, these findings require more extensive sample sequencing validation and subsequent studies using animal models and cell cultures. Most importantly, the microbiome’s complexity and the intricate relationships between microbial communities and cancer treatment responses are difficult to capture fully in studies with small sample sizes. Therefore, larger-scale, prospective studies are necessary to advance microbiome-based interventions against ESCC, emphasizing the need for robust, multi-center collaborations to enhance the understanding of the microbiome’s role in cancer therapy.

In summary, integrating machine learning algorithms like LightGBM into microbiome research enhances our ability to process complex data and identify predictive microbial markers. Our pioneering study demonstrates the significant predictive power of the gut microbiome for NICT responses in ESCC, achieving high accuracy in predicting patient outcomes. We identified dynamic changes in microbial communities, particularly SCFA-producing bacteria, as key biomarkers influencing treatment efficacy. This approach improves clinical decision-making and personalizes therapy strategies, highlighting the gut microbiome’s pivotal role in cancer therapy outcomes. Further research is essential to unravel the mechanisms behind these microbial indicators and refine microbiome-based interventions, promising innovative approaches to treatment customization in oncology. This ongoing inquiry advances our capability to optimize treatment strategies based on the intricate dynamics of the gut microbiome, setting the stage for a new era in oncology guided by microbiome-driven insights.

## Materials and Methods

### Research design and participant selection

In alignment with the 1975 Declaration of Helsinki, this retrospective cohort study was conducted at Nanfang Hospital, Southern Medical University. The study received ethical clearance from the Medical Ethics Committee of Nanfang Hospital (NFEC-2022-383). Prior to their participation, all individuals involved gave their written informed consent.

The study population consisted of 95 patients with ESCC undergoing NICT therapy, recruited from April 2020 to June 2023. Exclusion criteria encompassed the recent consumption (within 1 month prior to fecal sample collection) of antibiotics, prebiotics, probiotics, steroids, or immunosuppressants. Further details regarding the inclusion and exclusion criteria are available in the Supplementary Materials. After a thorough screening, 68 patients were deemed suitable for the study. To serve as a baseline comparison, 19 HCs without any malignancies, determined by clinical evaluation, were also included (Fig. 1A). These control participants were living in the same household as the ESCC patients to minimize variability due to lifestyle or dietary differences. NICT treatment involves at least 3 cycles of platinum combined with paclitaxel chemotherapy and PD-1 inhibitor (pembrolizumab, camrelizumab, tislelizumab, toripalimab, nivolumab, or sintilimab)-based immunotherapy. All patients underwent curative esophagectomy and lymphadenectomy within 4 to 6 weeks of completing NICT. This study meticulously collected demographic and clinicopathological information from participants. The AJCC’s (eighth edition) tumor regression grade (TRG) system, which has 4 levels, was utilized to assess responses to NICT therapy. This categorization led to the division of the ESCC cohort into 2 groups: 26 patients with TRG scores of 0 to 1 were identified as responders, whereas 42 with TRG scores of 2 to 3 were classified as nonresponders. Representative hematoxylin and eosin (H&E) staining images from both responders and nonresponders are presented in Fig. S1, illustrating the differences in tumor regression after NICT therapy. The methodology of the study is depicted in a flowchart provided in Fig. 1B.

### Sample collection and DNA isolation from feces

Following the procedure outlined in the Human Microbiome Project’s Manual of Procedures Protocol (#07-001), fecal samples were systematically collected. For each participant diagnosed with ESCC, a pair of fecal samples was obtained: one at the initiation of the study (before NICT therapy, *n* = 68) and another within a 3-day window following the completion of NICT therapy (after NICT therapy, *n* = 68). For the control group, consisting of healthy individuals, a single fecal sample was gathered during the initial phase of the study (HC, *n* = 19). Prior to analysis, all collected samples were preserved at a temperature of −80 °C. DNA was isolated from these samples utilizing the Bio-tek Stool DNA Kit (D4015, Omega Inc., USA), adhering strictly to the protocol provided by the manufacturer.

### PCR amplification and 16*S* rRNA sequencing

To investigate the hypervariable V3–V4 region of the 16*S* rRNA gene, we utilized modified primers 341F and 805R to amplify DNA isolated from fecal specimens. The polymerase chain reaction (PCR) protocol included an initial denaturation step at 98 °C for 30 s, followed by 32 cycles consisting of denaturation at 98 °C for 10 s, annealing at 54 °C for 30 s, extension at 72 °C for 45 s, and concluding with a final extension at 72 °C for 10 min. After amplification, the PCR products underwent purification using AMPure XT beads (Beckman Coulter Genomics, Danvers, MA, USA) and were quantitatively analyzed with the Qubit system (Invitrogen, USA). Sequencing was carried out on an Illumina NovaSeq 6000, facilitated by LC-Bio (Hangzhou, China), ensuring high-throughput and accurate data generation. The collected raw sequence data have been responsibly archived in the Genome Sequence Archive of the National Genomics Data Center, providing a valuable resource for future genomic studies.

### Sequence processing and bioinformatics analysis

Our bioinformatics workflow commenced with the integration of paired-end raw sequences through FLASH (version 1.2.8), followed by quality assurance and cleaning via fqtrim (version 0.9.4), ensuring high-quality sequence data for analysis. Chimeric sequences were identified and removed, and amplicon sequence variant (ASV), along with its representative sequences, was delineated using Vsearch (version 2.3.4). These sequences were then aligned against the Ribosomal Database Project classifier to obtain precise taxonomic annotations.

To accommodate variations in sample size, we normalized the data, setting the stage for comprehensive diversity analysis. Using R version 4.0.3 (including vegan, ggplot2, and ade4), we calculated both alpha and beta diversity metrics. Alpha diversity, indicating the variety within individual samples, was quantified using indices of richness (Chao1), abundance (Shannon), and a combined measure of richness and evenness (Simpson). Beta diversity metrics, on the other hand, assessed differences between groups, employing UniFrac for phylogenetic insights and Jaccard and Bray–Curtis distances for taxa abundance and presence analysis.

For a holistic view of microbial community structures, we conducted PCoA using the aforementioned beta diversity distances. Taxonomic abundance was scrutinized using Welch’s *t* test through STAMP (version 2.1.3). Identification of discriminatory taxa between groups utilized LEfSe, setting the linear discriminant analysis (LDA) score threshold at 3.0 to ensure meaningful differentiation. In exploring microbial interactions, co-occurrence networks were inferred through Spearman’s rank correlations and visualized in R software (version 4.0.3), adhering to stringent significance (*P* < 0.05) criteria.

Lastly, microbial phenotypic predictions were undertaken using BugBase, offering insights into the functional capabilities of the microbial communities under investigation. This comprehensive approach not only delineates the complex microbial landscape but also illuminates potential pathways through which these communities influence health and disease.

### Statistical analysis

For continuous variables, we employed either Student’s *t* test, Welch’s *t* test, or Mann–Whitney test based on the distribution characteristics of the data. Categorical variables, on the other hand, were evaluated using either Pearson’s chi-square test or Fisher’s exact test, ensuring appropriate analysis based on the expected frequencies within the data. To assess the statistical significance of beta diversity dissimilarities among predefined groups, we utilized permutational multivariate analysis of variance (PERMANOVA). All statistical analyses were meticulously carried out within the R software environment, adhering to a 2-sided *P*-value threshold of 0.05 to delineate statistical significance.

### Development of a response prediction model

To establish a classifier capable of predicting therapeutic responses, we implemented a multifaceted machine learning approach. Initially, the light gradient boosting machine (LightGBM) framework was employed for biomarker identification and classifier construction. We partitioned participants into training and testing cohorts at a 4:1 ratio, ensuring a robust validation of our predictive model. Through the application of XGBoost-RFE [52], we meticulously selected predictive biomarkers. The classifier’s efficacy was rigorously tested through various metrics, including ROC curves, alongside evaluations of accuracy, precision, recall, and the F1 score [53].

Furthermore, we integrated SHAPs for both local and global model interpretation, leveraging its game-theoretical underpinnings [26]. SHAP values offer a profound insight into feature importance and their impact on model predictions, providing an interpretative advantage through force plots. These plots elucidate the influence of individual features on specific predictions, enhancing our understanding of the model’s decision-making process. Notably, SHAP’s ability to elucidate feature importance and its predictive distribution ensures a transparent and trustworthy analytical framework. This comprehensive approach not only enhances the model’s predictive accuracy but also fosters trust and interpretability in its outcomes.

## Data Availability

ata are available on reasonable request. Further information and requests for resources and reagents should be directed to the lead contact S.M. (drmaishijie134@gmail.com).
