# Supplementary material for: Unveiling the Power of Gut Microbiome in Predicting Neoadjuvant Immunochemotherapy Responses in Esophageal Squamous Cell Carcinoma
Source: Research (Wash D C). 2024 Nov 14;7:0529. doi: 10.34133/research.0529 (PMC11562848; doi:10.34133/research.0529)
Supplement: Supplementary 1 — Supplementary Methods Figs. S1 to S3 [file research.0529.f1.zip › Supplementary Figures.docx]

**Supplementary Figures**

**Supplementary Fig. S1:** Histopathological comparison of ESCC tissue responses to NICT. H&E staining of ESCC tissues from two patients classified based on their response to NICT therapy (scale bar=50 μm).

**Supplementary Fig. S2:** Alterations in gut microbiome diversity across different response states to NICT therapy. Alpha diversity was estimated using the observed OTUs (A, D), Chao1 (B, E), and Shannon Diversity Index (C, F), and analyzed by Wilcoxon rank sum test and represents P<0.05. Beta diversity analysis was performed by using the Bray_Curtis (G, J), Jaccard (H, K), and weighted UniFrac (I, L), -based PCoA.
