# Supplementary material for: Unveiling the Power of Gut Microbiome in Predicting Neoadjuvant Immunochemotherapy Responses in Esophageal Squamous Cell Carcinoma
Source: Research (Wash D C). 2024 Nov 14;7:0529. doi: 10.34133/research.0529 (PMC11562848; doi:10.34133/research.0529)
Supplement: Supplementary 1 — Supplementary Methods Figs. S1 to S3 [file research.0529.f1.zip › Supplementary Materials.docx]

**Supplementary Methods**

**Inclusion criteria**

**For this study on esophageal squamous cell carcinoma (ESCC), we are seeking participants who align with the following criteria:**

1. Individuals fully informed about the study who have provided written consent;
2. Aged between 18 and 80;
3. Eastern Cooperative Oncology Group (ECOG) performance status of 0 or 1;
4. Histological confirmation of ESCC;
5. Absence of metastatic lesions as confirmed by imaging studies;
6. Deemed suitable for neoadjuvant immunochemotherapy (NICT) based on a thorough evaluation by our team;
7. Sufficient organ functionality is required to endure the treatment and associated study protocols, with test outcomes falling within acceptable parameters: hemoglobin levels should be no less than 90 g/L, neutrophil count above 1.5 x 10^9/L, and platelet count exceeding 100 x 10^9/L. Additionally, liver function indicators (transaminases up to 2.5 times the normal upper limit, and total bilirubin not surpassing 1.5 times the upper limit) and kidney function (serum creatinine should not exceed 1.5 times the upper limit) must be within the normal range.

**For healthy individuals, those who meet all the following inclusion criteria will be enrolled into this study:**

1. Provide informed consent after fully understanding the study's scope;
2. Aged between 18 and 80;
3. Be evaluated by the research team as free from any malignancies, based on a thorough review of clinical symptoms, physical examinations, laboratory tests, and imaging studies;
4. Must possess test outcomes that are within the normal limits for multiple evaluations, including blood, urine, and fecal tests, along with biochemical analyses.

**Exclusion criteria**

**For our study involving both ESCC patients and healthy volunteers, individuals will be excluded if they meet any of the following criteria:**

1. Previous treatment with radiotherapy;
2. Inability to complete three cycles of NICT;
3. A history of other malignancies in the past 5 years;
4. Presence of metabolic diseases, such as diabetes;
5. Undergone abdominal surgery that could impact the microbiome analysis;
6. Currently suffering from a severe active infection, including COVID-19;
7. History of infections including HIV, syphilis, or tuberculosis;
8. Recent contact (within one month prior to fecal sample collection) with any anticancer therapies or radiation treatments.
9. Administration of antibiotics, prebiotics, probiotics, steroids, or immunosuppressants within one month before collecting fecal samples.
